# Food-Loss Control at the Macronutrient Level: Protein Inventory for the Norwegian Farmed Salmon Production System

**DOI:** 10.3390/foods9081095

**Published:** 2020-08-11

**Authors:** Mohd Abualtaher, Eirin Skjøndal Bar

**Affiliations:** Department of Biotechnology and Food Science, NTNU-Norwegian University of Science and Technology, 7491 Trondheim, Norway; eirin.bar@ntnu.no

**Keywords:** responsible seafood producer, aquaculture, Atlantic salmon (*Salmo salar*), substance flow analysis (SFA)

## Abstract

The growing world population and the growing need for food are raising the importance of more efficient and sustainable food production systems. Food loss is a significant global challenge and a major stressor on natural resources. True assessment of food loss is a precursor to its reduction. This study aimed to assess the actual food loss in the Norwegian farmed salmon production system in the year 2019 by quantifying the protein flows and stocks in the system. Protein served as an indicator substance of the true systemic food loss. This study highlights the system’s qualitative value-adding conversion of plant protein into higher quality marine animal protein, with deposited vital trace minerals harvested from the sea and carried to the human food chain. However, it takes a lot of protein from multiple sources to produce salmon. We found that the total invested feed protein is about four times more than the harvested salmon protein, and about 40% of that harvested protein in the salmon biomass departs the human food chain by flowing to other non-food industries. The current post-harvest practices, material trade-offs, and waste management solutions could be adjusted to a context that prioritizes human food security. An alternative scenario is presented in this study, based on a hypothetical new food product in parallel to the main salmon fillet product. The alternative scenario turned 99% of the harvested protein into food and adjusted the ratio between the invested marine protein and the human food product protein. The originality of this research is in its approach to food loss assessment at the industrial level by means of a systemic macronutrient (protein) inventory.

## 1. Introduction

The Malthusian dilemma of a growing population versus the Earth’s carrying capacity is gaining momentum [1,2]. In a reality where hunger and malnutrition are the biggest risks to health worldwide, affecting hundreds of millions of people [3], the total population of 7.7 billion in 2019 is expected to reach 9.7 billion in 2050 [4]. Hunger and malnutrition are consequences of unsustainable food systems [5]. The United Nations 2030 Agenda for Sustainable Development clearly pointed toward the global challenge of developing sustainable food systems with set goals to be pursued. Sustainable Development Goal (SDG) 2 raised the issues of hunger, malnutrition, food access, and affordability, while SDG 12 addressed responsible food production and consumption through reducing food loss (FL) and food waste (FW) [6]. The United Nations Food and Agriculture Organization (FAO) clearly defines the technical term FL as the decrease in the quantity or quality of food resulting from decisions and actions by food suppliers in the chain before the retail stage. After it reaches retailers and consumers, it is described as FW [7,8]. A clear distinction in terminology draws borders of responsibility between industry versus retailers and producers versus consumers. FLs are taking place in various food supply chains at the different stages of production, post-harvest processing, and distribution. It is critical to assess the extent and causes of FL at the industrial level with consideration of the effectiveness and feasibility of its prevention and reduction measures. Developing appropriate strategies to reduce FL and FW is one of the most important issues in relation to sustainable development [9].

FL is causing major negative impacts on the environment, food security, and livelihoods of economically vulnerable people. Therefore, reducing FL to a minimum is vital for the sustainability of food supply chains. Reducing FL starts with the accurate assessment of the quantities of lost food [10]. For this purpose, assessments using qualitative and quantitative methods interactively have been developed and applied. The level of FL differs from one stage in the food supply chain to another, which raises the importance of an integrated, holistic approach to assess FL and to define its causes [11]. The various food production systems have different kinds of FL specific to the kind of harvested biomass, be it is plant crops, animals, dairy, fisheries, or aquaculture; therefore, methods need to be considerate of the technical specificities of production systems and processed materials. Food processing is the stage at which most FL is taking place, far more than the supporting logistical operations [12]. FL is commonly referred to as the decrease in the quantity of the “edible parts” of plants and animals that are produced or harvested for human consumption, but that are not ultimately consumed by people [13]. The description of edible parts is too supple, and is influenced by the product design, market preferences, culinary culture, processing costs, and revenue [14]. Post-harvest processing by-products are the subject of innovation and development for new food products in many different food value chains around the globe [15,16,17,18,19,20].

In this study, we approached FL in one of the global seafood supply chains with a tailored method for this purpose. The Norwegian farmed salmon industry is a globally high-profile seafood supplier, a significant economical contributor, and a steadily growing aquaculture producer [14,21,22]. Total global production of farmed Atlantic salmon in 2019 reached 2.6 million tons, 66% of which was produced in Norway [23]. Besides Norway, farmed Atlantic salmon is produced in Chile, Scotland, Canada, the Faroe Islands, and Sweden, and is just starting up in a few other countries. The major markets for farmed Atlantic salmon are Japan, the European Union, China, and North America.

Norway, the world’s largest producer of Atlantic salmon, started salmon farming 50 years ago, persevering with work in research and development. The industry successfully achieved breakthroughs at all levels—i.e., technological, economic, and environmental. The research was fruitful in developing salmon feed, feed technology, aquaculture equipment, vaccines, and fish farming management methods [24].

The sustainability endeavor in the Norwegian farmed salmon industry is rising as a result of the government’s regulated commitment [25], industrial interests, and perseverance in scientific research [26]. The main limitations and challenges facing the Norwegian salmon aquaculture are sea lice parasites [27] and fish escapees [28], for both ecological and operational reasons. The published scientific literature from Norwegian institutions and corporate sustainability reports reflects seeking sustainability for the salmon value chain in a holistic and inclusive manner [26,28,29,30]. The aspects of resource conservation, ecological impact, and climate change are frequently present in their measurable parameters. However, the FL issue is embodied in multiple contexts of material efficiency, feed raw material development, biological feed conversion rates, and the utilization of the rest of the raw material. Therefore, an FL assessment for the Norwegian farmed salmon supply chain is a fertile area for further investigation and for the introduction of approaches and methods developed for that purpose.

FL in fish value chains is a globally recognized significant target [8,21]. Achieving FL reduction requires qualitative decisions on which material is food and which is not as a starting point. This is justified by the fact that significant portions of harvested biomass are usually classified inedible or excluded as non-food material in different production systems for various reasons. Material within the food production systems, especially at post-harvest processing, is classified according to the design of the main product, the marketing desirability, the culinary culture, the quality control standards, and several other perspectives. To tackle this issue, we considered the material’s nutrient content as the major criterion that is more objective in judging its potential and value as food. In fish value chains, the estimated weight of post-harvest processing by-products (heads, viscera, frames, skins, tails, fins, scales, blood, etc.) is between 50% and 70% of the whole fish [31], which is usually classified inedible.

Atlantic salmon (*Salmo salar*) can be described as nutrient-dense biomass that contains high-quality marine animal protein, Omega-3 fatty acids i.e., eicosapentaenoic acid (EPA), and docosahexaenoic acid (DHA), vitamins (i.e., A, D, Niacin, and B12), and vital trace minerals (i.e., selenium, phosphorous, potassium, magnesium, zinc, iodine, and calcium) [32,33]. It is no wonder that salmon tissue carries all these trace minerals when grown in sea cages in a sea water medium. Salmon’s desirable pink color comes from a pigment called astaxanthin, a natural antioxidant compound, with proven health benefits [34]. Protein is a structural macronutrient that is clustered with all of the other micronutrients that are vital for human nutrition [35]. The role of protein in nutrition as a provider for adequate amounts of necessary amino acids, as well as its quality, is determined by its content of amino acids. Salmon protein is a high-quality marine protein, containing all of the essential amino acids [36]. Protein is the major component of salmon feed pellets; however, salmon feed is made with a mixture of plant protein and marine fish protein. With research on and the development of salmon feed, the percentage of plant protein ingredients (mainly soy and other grains) is increasing, while the amount of marine protein is decreasing. The marine protein feed ingredient is harvested from multiple fish species (e.g., anchovies, herring, sardines, and blue whiting), and it is still a major component of salmon feed pellets [37,38].

Salmon in Norway are grown in sea cages, which is a system that has many advantages over land-based aquaculture. For instance, there is no need for water filtration and the sea water can contribute to the nourishment of salmon with its rich content of minerals and electrolytes. However, in repeated incidents, fish escape from sea cages to the surrounding environment. Furthermore, fish excretions mixed with the spilled feed sit at the bottom of the cage as a sludge material, which can cause environmental problems such as eutrophication [39]. To tackle this problem, experiments have been done using sludge for energy production (biogas) and fertilizers [40].

At the post-harvest processing stage, where the salmon fillet is the targeted main product, all the processing by-products are transferred to the ensiling process, an enzymatic hydrolysis in acidic conditions to break down the tissue, which limits the growth of spoilage bacteria. The ensiling process flows from the reception of the rest of the raw material, through the physical treatment (mincing/grinding), addition of acid, mixing, hydrolysis, and heat treatment, to oil recovery and storage. The resulting silage is a thick liquid that is used for several other purposes, such as fur animal feed [40,41].

The ensiling of salmon by-products reduces the overall quality of the material in general, thus losing all of its sensory and texture qualities as seafood. Moreover, the protein quality is severely reduced and might cause its total degradation to a non-protein nitrogen compound if the hydrolysis process is not stopped at the right time [41]. The estimated protein recovery after the hydrolysis process ranges between 47% and 70% of the starting protein content of salmon by-product material [42]. Earlier studies found that soluble and insoluble protein isolates from salmon head and viscera have potential as functional protein ingredients [43,44].

In general, there are proven huge potential benefits from the utilization of salmon aquaculture by-products, such as environmental, economic, and food security [45]. This potential needs to be explored and presented to the industry decision-makers and developers.

Applying the concept of defining salmon by-product materials by their nutrient load and not by market-based edibility classifications [14], in this study we chose protein as an indicator substance of actual FL. By assessing the amount of protein being lost from the human food chain, we can establish clear insight into the amount of real FL independent of main product design, market bias, or customary food perceptions.

The available information and measurements on Norwegian farmed Atlantic salmon (*Salmo salar*) anatomy, tissue chemical composition, feed protein content, and biological conversion rates were all employed in this study to account for the fractioning of the material and its protein load through all stages of production. Applying a protein inventory to the system can reveal the true nutrients cost of production and the potential value of salmon by-products as a food and a rich source of nutrients. The results are discussed to identify the reasons for FL and the possible measures for its reduction through a suggested alternative scenario.

This study aimed to contribute to the discussion on farmed salmon value chain sustainability and FL reduction by investigating the following:

(1) The quantities of input and output protein for every process.
(2) The potential of salmon by-products as food and the amount of protein that does not reach the human food chain.
(3) How the system’s protein inventory can more accurately reflect the true FL and can assess the efficiency of biomass conversion into food.

## 2. Methods

For the purpose of establishing a protein inventory by mapping the protein flows and quantities in each process, we followed the basic procedures of the method of substance flow analysis with the adjustments highlighted and explained in the discussion

### 2.1. Substance Flow Analysis (SFA)

SFA is an analytical method of tracing and quantifying flows and stocks of a specific substance within a well identified system [46]. SFA can be effectively used to asses and support sustainable development of the industry [26,47]. It is a key tool for effective resource management, as well as for investigating and quantifying secondary or anthropogenic resource stocks [48].The SFA method includes the following basic steps: (1) Definition of research objective and selection of monitoring indicators; (2) system definition, including scope, boundaries, and time frame; (3) identification of relevant flows, processes, and stocks; (4) design of substance flow chart; (5) mass balancing; and (6) illustration and interpretation of results and conclusions [2,46,47]. This method facilitates the creation and comparison of alternative scenarios [49].

### 2.2. System Definition and Flow Description

The investigated system is the Norwegian farmed Atlantic salmon (*Salmo salar*) production system based on in the harvest of year 2019. It is structured on three subsystems, namely, feed production, aquaculture, and post-harvest processing [14], and is tailed by uses and waste management solutions of the rest of the raw material. This study defined FL assessment as a research objective and selected protein as an indicator substance. A mass balanced protein flow analysis model was developed based on the existing practices and technology. In the interpretation and discussion of the results, consideration was given to (1) the differences at the substance level between plant and animal protein; (2) the spatial and temporal variations between the three sub systems (i.e., feed production, aquaculture, and post-harvest processing); and (3) fish nitrogenous secretions are not considered protein in terms of the definition of substances, although they result from protein metabolism, an unavoidable biological process, so they were not included in the model.

### 2.3. Data Sources and Quantification Method

Quantification of the flows according to the rate of annual production, the chemical composition of the feed, the feed-to-fish conversion rate, and the post-harvest processing outcomes were fractioned according to the anatomy of Atlantic salmon and the protein content. The quantities of produced salmon were sourced from the National Statistical Institute of Norway (Sbb.no), the main producer of official statistics in Norway. The amount of salmon produced by Norwegian aquaculture in 2019 totaled 1,357,304 tons. The calculations were based on equations, ratios, and cofactors obtained from published articles of scientific research and industrial corporate reports from Norway. The quantity of salmon feed consumed in 2019 was 1,726,297 tons [50]. To calculate the amount of spilled feed (estimated to be 368,993 tons), we deducted the amount of eaten feed from the total consumed feed using the reported biological feed conversion ratio (Bio-FCR), estimated to be 1.0 (1 kg of feed to produce 1 kg of salmon) for eaten feed [51].

The feed protein content was 54.7%, split into 14.5% for marine protein and 40.2% for plant protein [51]. Escaped salmon from cages in 2019 reported by the Norwegian directorate of fisheries equaled 284,308 fish [52], with an assumed average weight of 2.7 kg per fish, totaling approximately 767.6 tons.

Atlantic salmon body parts, their weight fractions, and the protein content of each portion were used to calculate the quantities of protein in the post-harvest outputs and by-products (Table 1).

By-product utilization technology reports on the quantity of farmed Salmon by-product utilization from the previous year (2018) mentioned that 90% of the material was used for other industries. The used by-products were divided as follows: 27% for biogas/energy, 24% for livestock fodder, 23% for fish feed, 17% for the pet food industry, 2% for fur animal feed, and 7% for miscellaneous uses [58,59]. There were no reports of change in by-product flows in 2019.

### 2.4. Variability Margins and Uncertainties

SFA models come with a level of uncertainty due to the variability of data sources [26]. We gathered the data and all information from Norway and salmon-specific reliable sources as an approach to minimize uncertainties. We applied the Hedbrant and Sörme method for determining uncertainty [60] by allocating the level of uncertainty to the reliability and accuracy of the information source. The equations used to construct this model were obtained from peer-reviewed published scientific research papers. Data on the production quantity of salmon were sourced from published official statistics collected on the local, regional, and national levels. With 95% probability, all sources of information in this study retained uncertainty levels of 0 and 1, as shown in Table 2.

The intervals were inserted in the following equation to calculate the uncertainty factor:

F_a.b_ = 1 + [(F_a_ − 1)^2^ + (F_b_ − 1)^2^]^0.5^ (1)

For example, the quantity of harvested salmon reported in the official statistics for 2019 was 1,357,304 tons (uncertainty interval*/1). The protein content in Atlantic salmon whole fish is 0.169 of its whole weight, as per the literature (uncertainty interval*/1.1). The calculated total harvested protein equaled 229,384.376 tons.

1 + [(1 − 1)2 + (1.1 − 1)2]^0.5^ = 1.1 (2)

Therefore, the amount of harvested salmon protein in 2019 was very likely between 252,322.80 and 208,531.25 tons.

Another example of calculating the quantity of protein in the total harvested salmon viscera = harvested salmon × weight fraction × protein content:

1,357,304 tons × 0.14 × 0.1 = 19,002.256 tons(3)

with the calculated uncertainty interval of:

1 + [(1 − 1)2 + (1.1 − 1)2 + (1.1 − 1)2]^0.5^ = 1.14 (4)

Therefore, the value likely falls between 16,668.646 tons and a maximum of 21,662.572 tons, with an average value of 20,332.414.

## 3. Results

### 3.1. Current Practices and Flows

**Figure 1:**
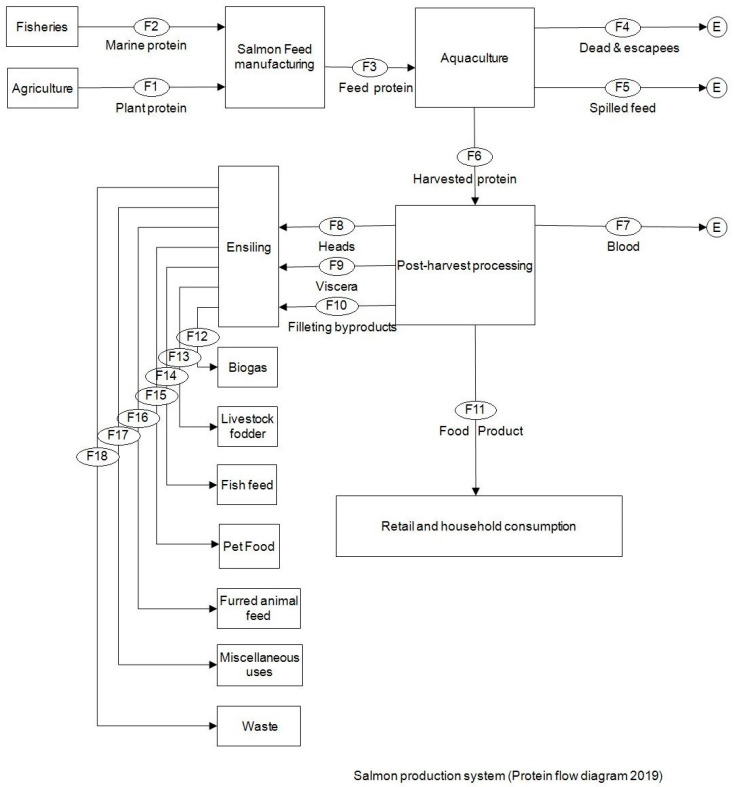
Processes and flows map.

A map for the farmed salmon production system with all protein flows according to current practices is shown in Figure 1. The quantities of protein are indicated in Table 3 for each flow.

**Figure 2:**
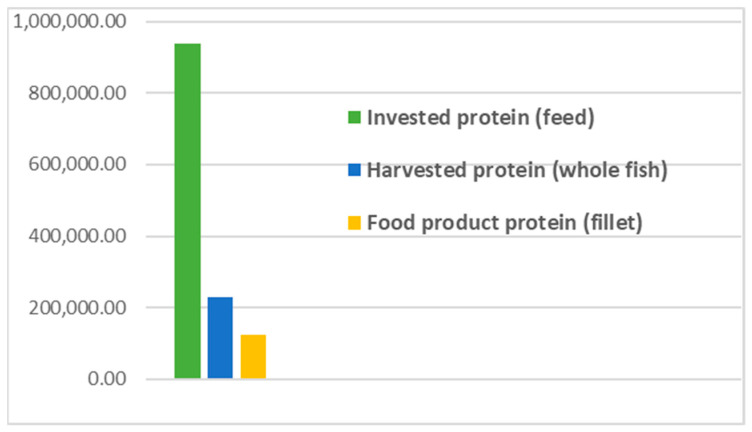
Quantity comparison.

**Figure 3:**
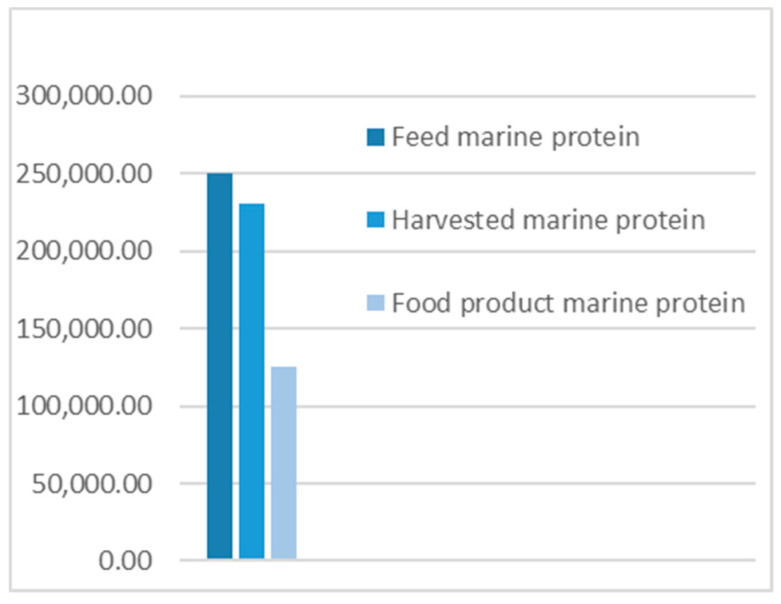
Marine animal protein quantities.

**Figure 4:**
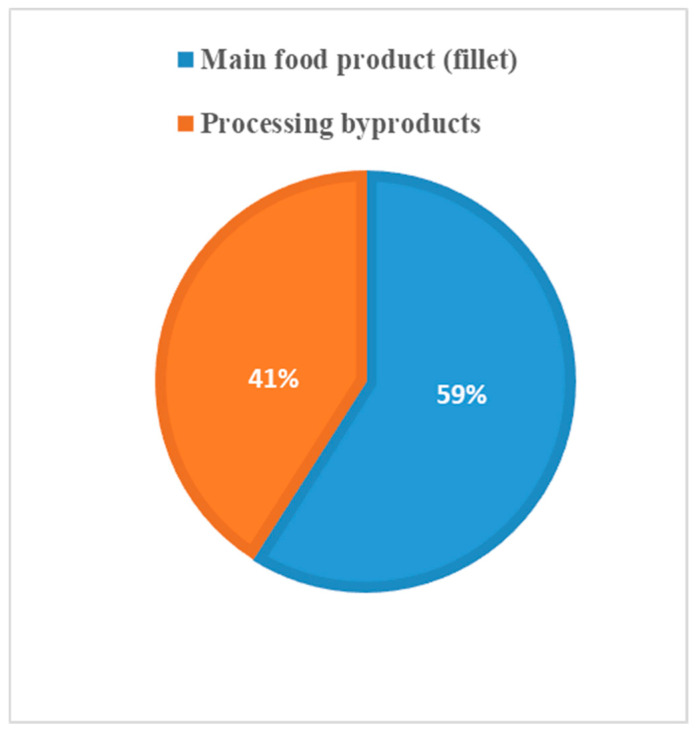
Post-harvest protein distribution.

Comparisons were made between the different flows and the quantities of protein, illustrated in Figure 2, Figure 3 and Figure 4.

### 3.2. Alternative Scenario

**Figure 5:**
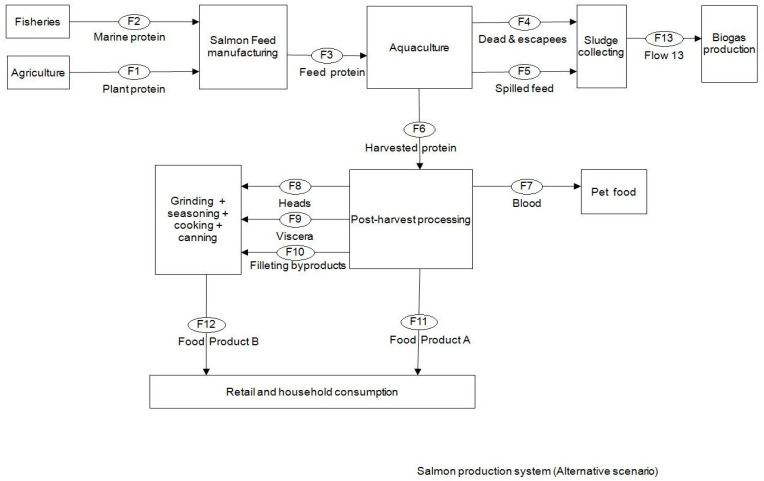
Alternative scenario.

**Figure 6:**
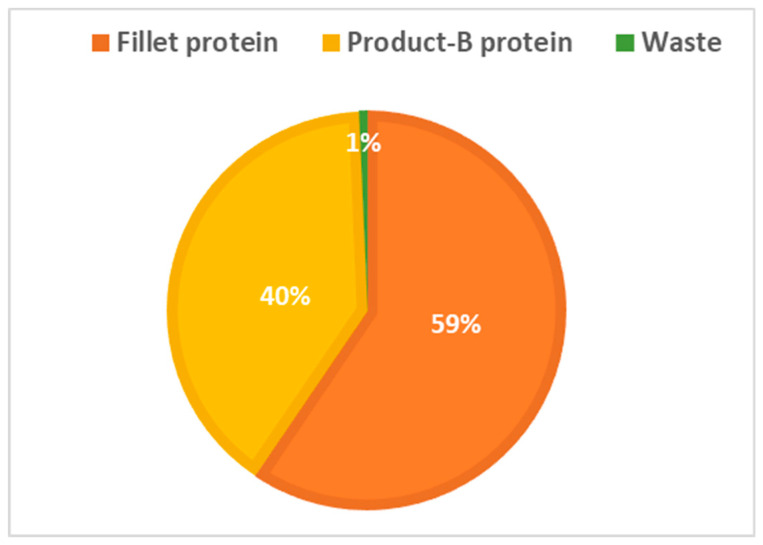
Alternative scenario post-harvest protein distribution.

**Figure 7:**
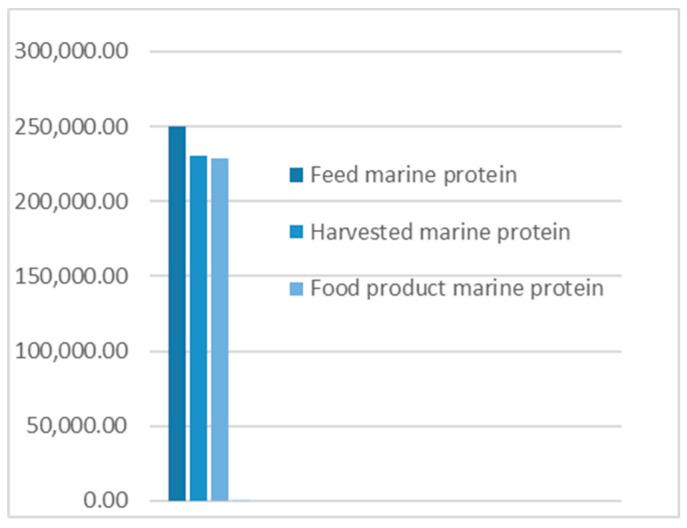
Alternative scenario marine protein quantities.

An alternative scenario was developed on the basis of conserving the harvested salmon protein and its holding biomass into the human food chain. This scenario limits all post-harvest flows into food production (Figure 5), presenting a need for a parallel food product B to be developed. Material trade-offs with pet food and biogas industries still exist, but in a different sequence. The change in the protein quantities in different flows suggested by this scenario illustrated in Figure 6 and Figure 7.

## 4. Discussion

- **The results** from applying protein SFA provided a clear picture about the system and the material. The total harvested salmon protein equaled less than 25% of the invested feed protein (Figure 2). The marine protein ingredient of the feed pellets equaled 10% more than the total harvested salmon protein (Figure 3). Approximately 41% of the total harvested salmon protein departed the human food chain for good as silage used for other industries (Figure 4). The estimated 85 kilotons of protein that turned into silage was embodied in approximately 650 kilotons of biomass. The quantity of protein in the model can reflect how nutrient-costly it is to produce salmon and how much of that salmon biomass is being lost during post-harvest processing.
- **The method** of SFA modeling of the quantity of protein flowing through the farmed salmon value chain served the objectives of FL assessment and reduction. Targeting protein, a biomolecule and a macronutrient, as an indicator substance provided a qualitative description of the material based on its optimum nutritional potential before the protein is broken down into other substances and nitrogen compounds. The method of substance flow analysis provided guidance on how to model a system and to quantify the flows and stocks of the traced substance; however, achieving the concept of 100% mass balance was not possible due to the complexity of biological processes and how the protein passes through fish metabolism, after which part of it is secreted as nitrogen compounds in feces and urine or breaks down during ensiling hydrolysis.
- **Uncertainties** of the SFA model result from seasonal and geographical variations, as this study included the three interlinked subsystems that structure this production system, and each subsystem has a level of independence, is geographically distant, and has operational needs and circumstances. We acknowledge the ambiguity due to variations in the size of the escaped fish, the number of dead and escapee fish, the amount of spilled feed, the seasonal variations in salmon physiology, and the percentage of protein content in the tissue. The available information and data covered most of the system and processes, with the exception of the miscellaneous uses of the silage (flow number 17). The reason for such a hazy description is that there are several experimental uses that are still under development.
- **The alternative scenario** in Section 3.2 (Figure 5) is based on further processing of the by-product material to re-introduce it as a marketable, quality food product that contains approximately 40% of the post-harvest protein (Figure 6). That will improve the system’s efficiency and bring more balance between invested and harvested marine protein (Figure 7) with increase in the protein quantity that becomes available for human consumers. In this alternative scenario, we highlighted the desirable impact of developing a new food product to be produced in parallel to salmon fillets, in a clear invitation to food scientists and product developers. The invested marine protein ingredient in salmon feed usually comes from anchovies and sardines, two fish species that are typically food-processed for canning. Creating a canned food product from the rest of the raw material of salmon provides a nutrient-dense, affordable sea food product with a long shelf life and that is ready to eat. Such a product would be a valuable compensation for the marine protein consumed as salmon feed. With the right recipe and a good marketing plan, the monetary revenue from turning the down-graded raw material remains to an affordable nutrient-dense seafood product would be substantial. This scenario, if materialized, would put an extra 650 kilotons of seafood on the global food table. The most significant limitation of this scenario is the quality status of the salmon by-products upon delivery for processing and their fitness for food ingredients. For this scenario to emerge and function, by-products need to reach the processing line with acceptable microbial load and free from spoilage. This raises the importance of localizing responsibility over salmon by-products on the same post-harvest processing facility for quick processing and for ensuring the freshness of the material, preceding microbial growth and rancidity. In this scenario, both the salmon fillet product and the hypothetical product B must come out of the same factory.
- **Other scenarios** based on dry matter extraction of protein and fat out of salmon by-products could be suggested. Macronutrient recovery from by-products would have a positive effect on the system’s efficiency and FL reduction “if” the recovered protein and fat were used as food. However, the total utilized biomass would be much less in comparison to the quantity in the first alternative scenario suggested by this study. Nutrient recovery requires further processing, associated with more production complications and costs. The main concept of this study was to consider macronutrients as indicators of the food material, not as a distinct target from the rest of the biomass. Our targeted FL reduction would be achieved if we were to manage to turn the whole 650 kilotons of salmon by-products into a food product, including but not limited to their 85-kiloton protein content.

## 5. Concluding Remarks

A system’s protein inventory can serve as a sustainability evaluation criterion for food production systems. It takes a lot of food/nutrients to produce a more expensive food like salmon. Sustainable and responsible seafood production demands seeking the optimum status of zero FL. The maximum utilization of harvested salmon biomass within the human food chain needs to be prioritized over any other non-food uses. The quantity of hidden FL in the farmed salmon production system is significant; thus, new food products need to be developed using salmon by-products as their main ingredient.

## Figures and Tables

**Table 1:** Protein content in Atlantic salmon body parts *.

Salmon Parts	Weight Fraction	Protein %
Whole fish	100%	16.9%
Blood	2%	5%
Viscera	14%	10%
Head	10%	15%
Filleting byproducts (i.e., backbone, belly bones, back fin, collarbone, tailpiece, belly flap, pin bones, fins, belly membrane, and skin)	24%	~12%
Fillet trims	2%	17%
Salmon fillet	48%	19%

* Sources: [41,44,53,54,55,56,57].

**Table 2:** Uncertainty intervals and data sources.

Level of Uncertainty	Interval	Source(s) of Information	Example(s)
0	*/1	Official statistics	Norway salmon production in 2019
1	*/1.1	Official statistics on local, regional, and national levels; values in general (from the literature); and information from the industry	Byproduct uses, protein content in salmon body parts, and feed content
2	*/1.33	Official statistics at the regional and national levels, and values in general (for content)	Monthly feed consumption in each region
3	*/2	Monitored data	Nitrogen compounds from protein hydrolysis
4	*/4	Values in general for flows (from the literature)	Sludge in fish cages

*/1, */1.1, */1.33, */2, */4—uncertainty intervals

**Table 3:** Calculated annual protein flows in 2019 *.

The Flows		Protein in Tons *		
Flow	Description	Minimum	Average	Maximum
F1	Plant protein feed ingredient	630,883.085	697,125.81	763,368.533
F2	Marine protein feed ingredient	227,557.332	250,313.065	275,344.372
F3	Feed pellets’ total protein content	847,454.891	936,437.655	1,025,420.418
F4	Dead/escaped salmon protein	114.03	131.12	148.2
F5	Spilled feed protein	174,786.16	200,969.13	227,152.1
F6	Harvested salmon protein (whole fish)	208,531.25	230,427.025	252,322.80
F7	Blood content of protein	1190.617	1368.972	1547.326
F8	Head	16,866.67	19,393.29	21,919.92
F9	Viscera	16,668.646	20,332.414	21,662.572
F10	Filleting byproducts and trims (backbone, belly bones, back fin, collarbone, tailpiece, belly flap, pin bones, fins, tissues, belly membrane, and skin)	38,337.885	44,080.9	49,823.915
F11	Food product for the market	108,584.32	124,850.25	141,116.182
F12	Silage for biogas	17,737.69	20,394.8	23,051.91
F13	Silage for livestock fodder	15,766.84	18,128.71	20,490.58
F14	Silage for fish feed	15,109.89	17,373.35	19,636.81
F15	Silage for pet feed	11,168.18	12,841.17	14,514.16
F16	Silage for furred animals feed	1313.90	1510.73	1707.55
F17	Silage for miscellaneous uses	4598.66	5287.54	5976.42
F18	Waste from silage	7299.46	8392.91	9486.37

* See text for details on calculating the uncertainty intervals.
